# *In silico* cancer research towards 3R

**DOI:** 10.1186/s12885-018-4302-0

**Published:** 2018-04-12

**Authors:** Claire Jean-Quartier, Fleur Jeanquartier, Igor Jurisica, Andreas Holzinger

**Affiliations:** 1 Holzinger Group, Institute for Medical Informatics, Statistics and Documentation, Medical University Graz, Graz, Austria; 2 Institute of Interactive Systems and Data Science, Graz University of Technology, Graz, Austria; 3 Krembil Research Institute, University Health Network; Depts. of Medical Bioph. and Comp. Sci., University of Toronto; Institute of Neuroimmunology, Slovak Academy of Sciences, Toronto, Canada

**Keywords:** Cancer research, Computational biology, Cancer bioinformatics, Integrative analysis, *In silico* modeling, In vitro methods, In vivo techniques, *Ex vivo* systems, Tumor growth, Alternative animal experimentation, 3Rs

## Abstract

**Background:**

Improving our understanding of cancer and other complex diseases requires integrating diverse data sets and algorithms. Intertwining in vivo and in vitro data and *in silico* models are paramount to overcome intrinsic difficulties given by data complexity. Importantly, this approach also helps to uncover underlying molecular mechanisms. Over the years, research has introduced multiple biochemical and computational methods to study the disease, many of which require animal experiments. However, modeling systems and the comparison of cellular processes in both eukaryotes and prokaryotes help to understand specific aspects of uncontrolled cell growth, eventually leading to improved planning of future experiments. According to the principles for humane techniques milestones in alternative animal testing involve in vitro methods such as cell-based models and microfluidic chips, as well as clinical tests of microdosing and imaging. Up-to-date, the range of alternative methods has expanded towards computational approaches, based on the use of information from past in vitro and in vivo experiments. In fact, *in silico* techniques are often underrated but can be vital to understanding fundamental processes in cancer. They can rival accuracy of biological assays, and they can provide essential focus and direction to reduce experimental cost.

**Main body:**

We give an overview on in vivo, in vitro and *in silico* methods used in cancer research. Common models as cell-lines, xenografts, or genetically modified rodents reflect relevant pathological processes to a different degree, but can not replicate the full spectrum of human disease. There is an increasing importance of computational biology, advancing from the task of assisting biological analysis with network biology approaches as the basis for understanding a cell’s functional organization up to model building for predictive systems.

**Conclusion:**

Underlining and extending the *in silico* approach with respect to the **3Rs** for **replacement**, **reduction** and **refinement** will lead cancer research towards *efficient* and *effective* precision medicine. Therefore, we suggest refined translational models and testing methods based on integrative analyses and the incorporation of computational biology within cancer research.

## Background

Cancer remains to be one of the top causes of disease-related death. World Health Organization (WHO) reported 8.8 million cancer-related deaths in 2015 [1]. Around one out of 250 people will develop cancer each year, and every fourth will die from it [2]. WHO estimates the number of new cases will rise by ∼ 70% over the next twenty years. Despite decades of research [3], mortality rates and recurrence remain high, and we have limited options for effective therapies or strategies regarding cancer prevention.

Tumor cells exhibit chaotic, heterogeneous and highly differentiated structures, which is determinative to the lack of effective anticancer drugs [4]. For that matter, predictive preclinical models that integrate in vivo, in vitro and *in silico* experiments, are rare but necessary for the process of understanding tumor complexity.

A biological system comprises a multiplicity of interconnected dynamic processes at different time and spatial range. The complexity often hinders the ability to detail relationships between cause and effect. Model-based approaches help to interprete complex and variable structures of a system and can account for biological mechanisms. Next to studying pathological processes or molecular mechanisms, they can be used for biomarker discovery, validation, basic approaches to therapy and preclinical testing. So far, preclinical research primarily involves in vivo models based on animal experimentation.

**Fig. 1:**
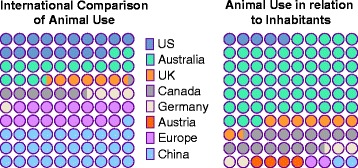
Worldwide use of animals for studies. International comparison in numbers of animals used for experimentation, such as toxicology testing for cosmetics, food, drugs, research, teaching and education [6–14]

Intertwining biological experiments with computational analyses and modeling may help to reduce the number of experiments required, and improve the quality of information gained from them [5]. Instead of broad high-throughput screens, focused screens can lead to increased sensitivity, improved validation rates, and reduced requirements for in vitro and in vivo experiments. For Austria, the estimated number of laboratory animal kills per year was over 200 000 [6]. In Germany the number of animal experiments for research is estimated as 2.8 millions [7]. Worldwide, the quantity of killed animals for research, teaching, testing and experimentation exceeds 100 000 000 per year [6–14], as shown in Fig. 1.

Principles for humane techniques were classified as replacement, reduction and refinement, also known as the 3Rs [15]. While most countries follow recommendations of Research Ethics Boards [16], discussion of ethical issues regarding the use of animals in research continues [17]. So far, 3R principles have been integrated into legislation and guidelines how to execute experiments using animal models, still, rethinking of refined experimentation will ultimately lead to higher-quality science [18]. The 3R concept also implies economic, ethical and academic sense behind sharing experimental animal resources, making biomedical research data scientifically easily available [19]. The idea behind 3R has been implemented in several programs such as Tox21 and ToxCast also offering high throughput assay screening data on several cancer-causing compounds for bioactivity profiles and predictive models [20–22].

It is clear that no model is perfect, and is lacking some aspects of reality. Thus, one has to choose and use appropriate models to advance specific experiments. Cancer research relies on diverse data from clinical trials, in vivo screens and validation studies, and functional studies using diverse in vitro experimental methods, such as cell-based models, spheroid systems, and screening systems for cytotoxicity, mutagenicity and cancerogenesis [23, 24]. New technologies will advance in organ-on-a-chip technologies [25] but also include the *in silico* branch of systems biology with its goal to create the virtual physiological human [26]. The range of alternative methods has already expanded further towards *in silico* experimentation standing for “performed on a computer”. These computational approaches include storage, exchange and use of information from past in vitro and in vivo experiments, predictions and modeling techniques [27]. In this regard, the term *non-testing* methods has been introduced, which summarizes the approach in predictive toxicology using previously given information for risk assessment of chemicals [28]. Such methods generate *non-testing* data by the general approach of grouping, (quantitative) structure-activity relationships (QSAR) or comprehensive expert systems, which are respectively based on the similarity principle [29–31].

The regulation of the European Union for registration, evaluation, authorisation and restriction of chemicals (REACH) promotes adaptation of in vivo experimentation under the conditions that *non-testing methods* or in vitro methods provide valid, reliable, relevant information, adequate for the intended purpose, or in case that testing is technically impossible [30].

Generally, in vitro and *in silico* are useful resources for predicting several (bio)chemical and (patho)physiological characteristics of likewise potential drugs or toxic compounds, but have not been fit for full pharmacokinetic profiling yet [32]. In vitro as well as *in silico* models abound especially in the fields of toxicology and cosmetics, based on cell culture, tissues and simulations [33]. In terms of 3R, in vitro techniques allow to reduce, refine and replace animal experiments. Still, wet biomedical research requires numerous resources from a variety of biological sources. *In silico* methods can further be used to augment and refine in vivo and in vitro models. Validation of computational models will still require results from in vivo and in vitro experiments. Though, in the long run, integrative approaches incorporating computational biology will reduce laboratory work in the first place and effectively succeed in 3R.

**Fig. 2:**
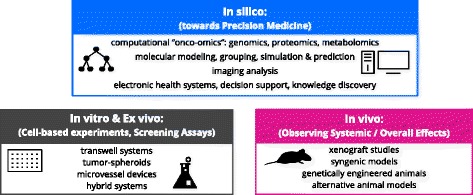
Preclinical techniques for cancer research. Examples for experiments on the computer (*in silico*), inside the living body (in vivo), outside the living body (*ex vivo*) as well as in the laboratory (in vitro)

Within the next sections, we summarize common methods and novel techniques regarding in vivo, in vitro and *in silico* cancer research, presented as overview in Fig. 2, and associated modeling examples listed in Table 1.

**Table 1:** Overview of exemplary models for cancer research

	References
In vivo	
Murine models	[37]
Genetically engineered mouse model	[36]
Zebra fish model	[42]
Drosophila model	[41]
Chick embryo model	[43, 44]
In vitro	
General 2D/3D in vitro models	[46, 53]
Transwell model	[48]
Spheroid system	[49]
Microfluidic system	[50]
Tissue-engineered microvessel model	[51]
In silico	
Sequence analysis	[63, 69, 74]
General pathway analysis and network inference	[132, 133, 135]
Pan-cancer	[62, 82, 134, 139]
Chemical perturbation mapping	[64, 66, 68]
Pharmacogenomic mapping	[99, 102, 117, 136]
Genome-phenotype mapping	[81]
Clinical data integration	[106]
Structure mapping	[102, 103]
Structure and activity	[100, 101]
Framework for key events and mode of action	[97, 98]
Image classification	[85, 87]
Growth prediction	[91–93]

## In vivo methods

Animals are the primary resource for research on the pathogenesis of cancer. Animal models are commonly used for studies on cancer biology and genetics as well as the preclinical investigation of cancer-therapy and the efficacy and safety of novel drugs [34]. Animal models represent the in vivo counterpart to cell-lines and suspension culture, while being superior in terms of physiological relevance offering imitation of parental tumors and a heterogeneous microenvironment as part of an interacting complex biochemical system.

In general, animal models primarily based on murine or rodent models can be subdivided into the following groups of (I) xenograft models, which refer to the heterotopic, subcutaneous intraperitoneal or orthotopic implantation into SCID (Severe Combined Immune Deficiency) or nude mice, (II) syngenic models involving the implantation of cells from the same strain into non-immunocompromised mice, and (III) genetically engineered models, which allow for RNA interference, multigenic mutation, inducible or reversible gene expression [35, 36].

Several engineered mouse models on cancer and related diseases have been developed so far [37]. In case of xenograft models, tumor-specific cells are transplanted into immunocompromised mice. Common tumor xenograft models lack the immune system response that can be crucial in tumor development and progression [38]. Xenograft models can be patient-derived, by transferation of a patient’s primary tumor cells after surgery into immunocompromised mice. The transplantation of immortalized tumor cell-lines represents a simplified preclinical model with limited clinical application possibilities [39]. For these reasons, there is a trend towards genetically engineered animal models, allowing for site-directed mutations on tumor-suppressor genes and proto-oncogenes as the basis for studies on oncogenesis [40].

Next to the gold standard of murine and rodent models, there are other animal model systems frequently used, such as the Drosophila melanogaster (fruit fly) or Danio rerio (zebra fish) [41, 42]. The fruit fly offers the advantage of low-cost handling and easy mutant generation while it holds a substantially high conservation of the human cancer-related signaling apparatus [41]. There are additional animal models, commonly referred to as alternatives, such as zebra fish models for angiogenesis studies and chick embryo CAM (chorioallantoic membrane) models, offering rapid tumor formation due to the highly vascularized CAM structure [40, 43, 44].

So far, preclinical model systems do not provide sufficient information on target validation, but aid in identifying and selecting novel targets, while new strategies offer a quantitative translation from preclinical studies to clinical applications [45].

## In vitro methods

In vitro models offer possibilities for studying several cellular aspects as the tumor microenvironment using specific cell types, extracellular matrices, and soluble factors [46]. In vitro models are mainly based on either cell cultures of adherent monolayers or free-floating suspension cells [47]. They can be categorized into: (I) transwell-based models which include invasion and migration assays [48], (II) spheroid-based models involving non-adherent surfaces [49], hanging droplets and microfluidic devices [50], (III) tumor-microvessel models which come with predefined ECM (extracellular matrix) scaffolds and microvessel self-assemblies [51], and (IV) hybrid tumor models including embedded *ex vivo* tumor sections, 3D invasion through clusters embedded in gel, and 2D vacscular microfluidics [52].

Generally, such cell culture models focus on key aspects of metabolism, absorption, distribution, excretion of chemicals or other aspects of cell signaling pathways, such as aspects of metastasis under a controlled environment [53]. Scale-up systems attempt to emulate the physiological variability in order to extrapolate from in vitro to in vivo [54]. Advanced models as 3D culture systems more accurately represent the tumor environment [55]. Cell culture techniques include the formation of cell spheroids, which are frequently used in cancer research for approximating in vitro tumor growth as well as tumor invasion [56]. In particular, multicellular tumor spheroids have been applied for drug screening and studies on proliferation, migration, invasion, immune interactions, remodeling, angiogenesis and interactions between tumor cells and the microenvironment [46].

In vitro methods include studies on intercellular, intracellular or even intraorganellar processes, which determine the complexity of tumor growth to cancerogenesis and metastasis, based on several methods from the disciplines of biophysics, biochemistry and molecular biology [23].

*Ex vivo* systems offer additional possibilities to study molecular features. Such systems can be derived from animal and human organs or multiple donors. Thereby, *ex vivo* systems comprise the isolation of primary material from an organism, cultivation and storage in vitro and differentiation into different cell types [57]. In this regard, induced pluripotent stem cells, in particular cancer stem cell subpopulations, have been presented as in vitro alternative to xenograft experiments [58]. Moreover, *ex vivo* methods can be used to predict drug response in cancer patients [59]. These systems have been developed to improve basic in vitro cell cultures while overcoming shortcomings of preclinical animal models; thus, serving as more clinically relevant models [60].

## *In silico* analysis

**Fig. 3:**
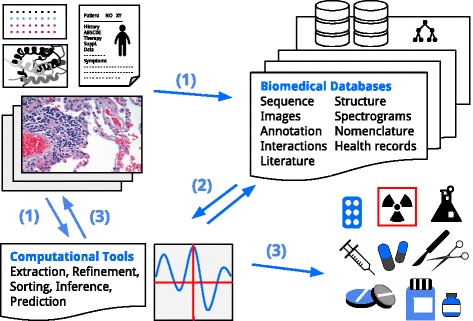
*In silico* pipeline. (1) Manual input into databases storing patient information, literature, images and experimental data, or direct data input into computational tools. (2) Refinement and retrieval over computational tools for classification, inference, validation and prediction. (3) Output for research strategies, model refinement, diagnosis, treatment and therapy. Note: Derivative elements have been identified as licensed under the Creative Commons, free to share and adapt

The term *in silico* was created in line with in vivo and in vitro, and refers to as performed on computer or via computer simulation [28]. *In silico* techniques can be summarized as the process of integrating computational approaches to biological analysis and simulation. So far, *in silico* cancer research involves several techniques including computational validation, classification, inference, prediction, as well as mathematical and computational modeling, summarized in Fig. 3. Computational biology and bioinformatics are mostly used to store and process large-scale experimental data, extract and provide information as well as develop integrative tools to support analysis tasks and to produce biological insights. Existing well-maintained databases provide, integrate and annotate ”information on various cancers [61], and are increasingly being used to generate predictive models, which in turn will inform and guide biomedical experiments. Table 2 lists several representative examples of such databases.

**Table 2:** List of main databases and data resources in cancer research

Name	Url	References
cBioPortal	http://www.cbioportal.org	[115, 127]
BioPreDyn-Bench	https://sites.google.com/site/biopredynbenchmarks/	[104]
CGAP	https://cgap.nci.nih.gov/	[62]
EPA Toxcast Screening Library	https://actor.epa.gov/dashboard/	[20, 65]
EpiFactors	http://epifactors.autosome.ru/	[76]
Human Protein Atlas	https://www.proteinatlas.org/	[110, 111]
GDSC	http://www.cancerrxgene.org/	[101, 103]
GDC/TCGA	https://portal.gdc.cancer.gov/	[61, 138]
Gene Ontology	http://www.geneontology.org/	[128]
KEGG	http://www.genome.jp/kegg/	[130]
NCI-60 databases	https://discover.nci.nih.gov/cellminer/	[115, 144]
Open TG-GATEs	http://toxico.nibiohn.go.jp/english/	[66]
Reactome	https://reactome.org/	[129]
pathDIP	http://ophid.utoronto.ca/pathDIP/	[134]

The Cancer genome project and Cancer Genome Atlas have generated an abundance of data on molecular alterations related to cancer [62]. The Cancer Genome Anatomy Project by the National Cancer Institute also provides information on healthy and cancer patient gene expression profiles and proteomic data with the objective to generate novel detection, diagnosis and treatment possibilities [63]. In this connection, analyzing molecular changes and collecting gene expression signatures of malignant cells is important for understanding cancer progression. As example, over a million profiles of genes, drugs and disease states have been collected as so-called cellular connectivity maps in order to discover new therapeutic targets for treating cancer [64]. Regarding the effect of small molecules on human health, computational toxicology has created *in silico* resources to organise, analyse, simulate, visualise, or predict toxicity as a measure of adverse effects of chemicals [31, 65]. Large-scale toxicogenomics data has been collected by multi-agency toxicity testing initiatives, for forecasting carcinogenicity or mutagenicity [20, 66–68]. Thereby, gene expression signatures and information on chemical pathway perturbation by carcinogenic and mutagenic compounds have been analyzed and incorporated into *in silico* models to predict the potential of hazard pathway activation including carcinogenicity to humans [20–22, 66].

The analysis of genomic and proteomic data largely focuses on comparison of annotated data sets, by applying diverse machine learning and statistical methods. Most genomic alterations comprise single nucleotide variants, short base insertions or deletions, gene copy number variants and sequence translocations [69]. Thereby, cancer genes are defined by genetic alterations, specifically selected from the cancer microenvironment, conferring an advantage on cancer cell growth. In this regard, the goal is set in characterizing driver genes. However, combination of such genes may provide prognostic signatures with clear clinical use. Integrating patterns of deregulated genome or proteome with information about biomolecular function and signaling cascades does in turn provide inside into underlying biological mechanism driving the disease.

Analysis of genomic and proteomic data relies on processing methods such as clustering algorithms [70]. Cluster analysis depicts the statistical process of group formation upon similarities, exemplary for exploratory data mining [71]. Understanding the heterogeneity of cancer diseases and the underlying individual variations requires translational personalized research such as statistical inference at the patient level [72]. Statistical inference represents the process of detailed reflections on data and deriving sample distributions, understanding large sample properties and concluding with scientific findings as knowledge discovery and decision making. This computational approach involving mathematical and biological modeling, allows to predict disease risk and progression [72].

Besides directly studying cancer genes and proteins, it is increasingly recognized that their regulators, not only involving so far known tumor suppressor genes and proto-oncogenes but also non-coding elements [73–75] and epigenetic factors in general can be highly altered in cancer [76, 77]. These include metabolic cofactors [78], chemical modifications such as DNA methylation [79], and microRNAs [80]. Another approach to studying cancer involves the view of dysregulated pathways instead of single genetic mutations [81]. The heterogeneous patient profiles are thereby analyzed for pathway similarities in order to define phenotypic subclasses related to genotypic causes to cancer. Next to elucidating novel genetic players in cancer diseases using genomic patient profiling, there are other studies focusing the underlying structural components of interacting protein residues in cancer [82]. This genomic-proteomic-structural approach is used to highlight functionally important genes in cancer. In this regard, studies on macromolecular structure and dynamics give insight into cellular processes as well as dysfunctions [83].

Image analysis and interpretation strongly benefit from diverse computational methods in general and within the field of cancer therapy and research. Computer algorithms are frequently used for classification purposes and assessment of images in order to increase throughput and generate objective results [84–86]. Image analysis via computerized tomography has been recently proposed for evaluating individualized tumor responses [87]. Pattern recognition describes a major example on extracting knowledge from imaging data. Recently, an algorithmic recognition approach of the underlying spatially resolved biochemical composition, within normal and diseased states, has been described for spectroscopic imaging [88]. Such an approach could serve as digital diagnostic resource for identifying cancer conditions, and complementing traditional diagnostic tests towards personalized medicine.

Computational biology provides resources and tools necessary for biologically-meaningful simulations, implementing powerful models of cancer using experimental data, supporting trend analysis, disease progression and strategic therapy assessment. Network models on cancer signaling have been build on the basis of time-course experiments measuring protein expression and activity in use of validating simulation prediction and testing drug target efficacy [89]. Simulations of metabolic events have been introduced with genome scale metabolic models for data interpretation, flux prediction, hypothesis testing, diagnostics, biomarker and drug target identification [90]. Mathematical and computational modeling have been further used to better understand cancer evolution [91–93].

Since the concept of 3R has its primary focus on replacing animal experimentation within the area of chemical assessment, several *in silico* methods have been or are being developed in the field of toxicology. So far, computational toxicology deals with the assessment of hazardous chemicals such as carcinogens rather than computational biomedicine and biological research concerning cancer. Still, underlying methods can be likewise integrated into both disciplines [94, 95]. Recently, toxicology has brought up the adverse outcome pathway (AOP) methodology, which is intended to collect, organise and evaluate relevant information on biological and toxicological effects of chemicals, more specifically, existing knowledge concerning biologically plausible and empirically supported links between molecular-level perturbation of a biological system and an adverse outcome at the level of biological organisation of regulatory concern [96, 97]. This framework is intended to focus humans as model organism on different biological levels rather than whole-animal models [95]. The International Program on Chemical Safety has also published a framework for analyzing the relevance of a cancer mode of action for humans, formerly assessed for carcinogenesis in animals [98]. The postulated mode of action comprises a description of critical and measurable key events leading to cancer. This framework has been integrated into the guidelines on risk assessment by the Environmental Protection Agency to provide a tool for harmonization and transparency of information on carcinogenic effect on humans, likewise intended to support risk assessors and also the research community. Noteworthy, next to frameworks, there are several common toxicological *in silico* techniques. Especially similarity methods play a fundamental role in computational toxicology with QSAR modeling as the most prominent example [28, 29]. QSARs mathematically relate structure-derived parameters, so-called molecular descriptors, to a measure of property or activity. Thereby, regression analysis and classification methods are used to generate a continuous or categorical result as qualitative or quantitative endpoint [29, 31]. Exemplary, models based on structure and activity data have been used to predict human toxicity endpoints for a number of carcinogens [22, 99–101]. Still, in order to predict drug efficacy and sensitivity, it is suggested to combine models on chemical features such as structure data with genomic features [102–104].

Combined, *in silico* methods can be used for both characterization and prediction. Thereby, simulations are frequently applied for the systematic analysis of cellular processes. Large-scale models on whole biological systems, including signal-transduction and metabolic pathways, face several challenges of accounted parameters at the cost of computing power [105]. Still, the complexity and heterogeneity of cancer as well as the corresponding vast amount of available data, asks for a systemic approach such as computational modeling and machine learning [106, 107]. Overall, *in silico* biological systems, especially integrated mathematical models, provide significant link and enrichment of in vitro and in vivo systems [108].

## Computational cancer research towards precision medicine

Oncogenesis and tumor progression of each patient are characterized by multitude of genomic perturbation events, resulting in diverse perturbations of signaling cascades, and thus requiring thorough molecular characterization for designing effective targeted therapies [109]. Precision medicine customizes healthcare by optimizing treatment to the individual requirements of a patient, often based on the genetic profile or other molecular biomarkers. This demands state-of-the-art diagnostic and prognostic tools, comprehensive molecular characterization of the tumor, as well as detailed electronic patient health records [110].

Computational tools offer the possibility of identifying new entities in signaling cascades as biomarkers and promising targets for anticancer therapy. For example, the Human Protein Atlas provides data on the distribution and the expression of putative gene products in normal and cancer tissues based on immunohistochemical images annotated by pathologists. This database provides cancer protein signatures to be analysed for potential biomarkers [111, 112].

A different approach to the discovery of potential signaling targets is described by metabolomic profiling of biological systems which has been applied to find novel biomarkers for detection and prognosis of the disease [113–115].

Moreover, computational cancer biology and pharmacogenomics have been used for gene targeting by drug repositioning [116, 117]. Computational drug repositioning is another example for *in silico* cancer research, by identifying novel use for FDA-approved drugs, based on available genomic, phenotypic data with the help of bioinformatics and chemoinformatics [118–120]. Computer-aided drug discovery and development have improved the efficiency of pharmaceutical research and link virtual screening methods, homology and molecular modeling techniques [121, 122]. Pharmacological modeling of drug exposures helps to understand therapeutic exposure-response relationships [123]. Systems pharmacology integrates pharmacokinetic and pharmacodynamic drug relations into the field of systems biology regarding the multiscale physiology [124]. The discipline of pharmacometrics advances to personalized therapy by linking drug response modeling and health records [125]. Polypharmacological effects of multi-drug therapies render exclusive wet lab experimentation unfeasible and require modeling frameworks such as system-level networks [126]. Network pharmacology models involve phenotypic responses and side effects due to a multi-drug treatment, offering information on inhibition, resistance and on-/off-targeting. Moreover, the network approach allows to understand variations within a single cancer disease regarding heterogeneous patient profiles, and in the process, to classify cancer subtypes and to identify novel drug targets [81].

Tumorigenesis is induced by driver mutations and embeds passenger mutations that both can result in upstream or downstream dysregulated signaling pathways [127]. Computational methods have been used to distinguish driver and passenger mutations in cancer pathways by using public genomic databases available through collaborative projects such as the International Cancer Genome Consortium or The Cancer Genome Atlas (TCGA) [62] and others [128], together with functional network analysis using *de novo* pathway learning methods or databases on known pathways such as Gene Ontology [129], Reactome [130] or the Kyoto Encyclopedia of Genes and Genomes (KEGG) [131–134]. These primary pathway databases, based on manually curated physical and functional protein interaction data, are essential for annotation and enrichment analysis. To increase proteome coverage of such analyses, pathways can be integrated with comprehensive protein-protein interaction data and data mining approaches to predict novel, functional protein:pathway associations [135]. Importantly, this *in silico* approach not only expands information on already known parts of the proteome, it also annotates current “pathway orphans” such as proteins that currently do not have any known pathway association.

Comprehensive preclinical models on molecular features of cancer and diverse therapeutic responses have been built as pharmacogenomic resource for precision oncology [136, 137]. Future efforts will need to expand integrative approaches to combine information on multiple levels of molecular aberrations in DNA, RNA, proteins and epigenetic factors [62, 138], as well as cellular aspects of the microenvironment and tumor purity [139], in order to extend treatment efficacy and further refine precision medicine.

## Conclusion

Informatics in aid to biomedical research, especially in the field of cancer research, faces the challenge of an overwhelming amount of available data, especially in future regards to personalized medicine [140]. Computational biology provides mathematical models and specialized algorithms to study and predict events in biological systems [141]. Certainly, biomedical researchers from diverse fields will require computational tools in order to better integrate, annotate, analyze, and extract knowledge from large networks of biological systems. This increasing need of understanding complex systems can be supported by “Executable Biology” [142], which embraces representative computational modeling of biological systems.

There is an evolution towards computational cancer research. In particular, *in silico* methods have been suggested for refining experimental programs of clinical and general biomedical studies involving laboratory work [143]. The principles of the 3Rs can be applied to cancer research for the reduction of animal research, saving resources as well as reducing costs spent on clinical and wet lab experiments. Computational modeling and simulations offer new possibilities for research. Cancer and biomedical science in general will benefit from the combination of *in silico* with in vitro and in vivo methods, resulting in higher specificity and speed, providing more accurate, more detailed and refined models faster. *In silico* cancer models have been proposed as refinement [143]. We further suggest the combination of *in silico* modeling and human computer interaction for knowledge discovery, gaining new insights, supporting prediction and decision making [144].

Here, we provided some thoughts as a motivator for fostering *in silico* modeling towards 3R, in consideration of refinement of testing methods, and gaining a better understanding of tumorigenesis as tumor promotion, progression and dynamics.

## Abbreviations

3R: Refinement, reduction, replacement
AOP: Adverse outcome pathway
CAM: Chorioallantoic membrane
ECM: Extracellular matrix
FDA: Food and drug administration
KEGG: Kyoto encyclopedia of genes and genomes
pathDIP: Pathway data integration portal
QSAR: Quantitative structure-activity relationship
REACH: Registration, evaluation, authorisation and restriction of chemicals
SCID: Severe combined immune deficiency
TCGA: The cancer genome atlas
WHO: World health organization
